# Stable isotopes from Earth science to bedside medicine: beyond tracers and toward mechanistic biomarkers in medical oncology

**DOI:** 10.1007/s12032-026-03436-x

**Published:** 2026-10-08

**Authors:** Emma U. Hammarlund, Per Malmberg, Julhash U. Kazi, Nicole R. Posth, Kenneth J. Pienta

**Affiliations:** 1 Tissue Development and Evolution (TiDE), Department of Experimental Medical Science, Lund University, Lund, Sweden; 2 Multi-elemental Insights of Origins (MIO) Consortium, Lund, Sweden; 3 Lund University Cancer Center (LUCC), Lund, Sweden; 4 Lund Stem Cell Center (SSC), Lund, Sweden; 5 Globe Institute, University of Copenhagen, Copenhagen, Denmark; 6 Department of Chemistry and Chemical Engineering, Chemistry and Biochemistry, Chalmers University of Technology, Gothenburg, Sweden; 7 Institute of Geology and Natural Resources, Department of Geosciences & Natural Resource Management (IGN), Copenhagen, Denmark; 8 Cancer Ecology Center, The Brady Urological Institute, Johns Hopkins School of Medicine, Baltimore, USA

**Keywords:** Stable isotopes, Reference materials, Isotope fractionation, Mechanistic biomarkers, Isotope metallomics, Mass spectrometry, Precisiononcology, Machine learning, Exposomics, Cancer

## Abstract

Stable isotopes are non-radioactive variants of the same element whose small mass differences produce predictable effects in chemical and biological systems. While medicine has mainly used the administration of enriched stable isotopes as tracers of physiological function, the natural sciences developed stable-isotope ratios into robust measures of source, transformation, and underlying mechanism across Earth and life history. This analytical power – that often goes beyond conventional concentration-based assays – emerged through advances in isotope theory, mass spectrometry, but perhaps most importantly also globally shared reference systematics. Here, we review that trajectory and argue that medicine is positioned for a similar shift, as new computational and analytical tools make isotope analysis increasingly compatible with clinical throughput. In this shift, some natural isotope variation may serve as mechanistic biomarkers of physiology and disease. Integrated with multiomics and machine learning, stable isotopes could support earlier detection, improved risk stratification, and personalized care, particularly in cancer.

## Introduction

Modern medicine rests on a multitude of measurements, but most of them are single snapshots. A blood- chemistry panel can reveal, for example, that concentrations of calcium or potassium are high or low at a particular moment. Yet such panels rarely reveal why those levels changed, what molecular pool they reflect, or what physiological process produced them. Stable (non-radioactive) isotopes can offer a complementary information about human biology beyond concentration measurements [1, 2]. Because isotopes of the same element differ slightly in mass, biological and chemical processes handle them differently – and those small biases show up as measurable isotope ratios [3]. Indeed, stable isotopes can report both process and origin in the same sample.

In the natural sciences, stable isotopes have made it possible to ask not only what is present, but why it is there and how it changed. The study of isotope ratios across compartments (or pools) became a tool to reconstruct past climates, map origins, and trace exposures, largely because the research community built shared scales and reference materials. The robustness of the method grew from, and allowed for, the comparison of results across laboratories and time. In parallel, the medical field pioneered the use of stable isotopes to trace function. Still today, tracer applications are used in, for example, gastroenterology, metabolic research and infectious disease [4–8]. However, the broader potential of natural-abundance isotope ratios as mechanistic biomarkers is still underutilized [9].

A distinction important for clinical readers is that enriched stable-isotope tracers require deliberate administration of a labelled substrate (e.g., ¹³C-urea, ¹³C-glucose, or deuterated water), whereas natural-abundance isotope-ratio biomarkers measure endogenous variation already present in tissue or biofluids without tracer administration [2, 9]. An example of the former is deuterium dilution for total body water, whereas geographic variation in the proportion of stable isotopes of oxygen (^16^O vs. ^18^O) or hydrogen (^1^H vs. ²H) in human tissues illustrates the latter [2, 10].

This review is written for medical oncologists, translational cancer biologists, and clinical researchers evaluating emerging biomarker modalities. We review how stable isotopes moved into scientific awareness and disciplines, from tracing presence to interpreting processes. We also argue for a next step that would bring the stable-isotope to the bedside, especially combined with computational methods. Such efforts could report not only what is present, but which processes are shifting in disease or treatment^4,9^. Stable isotope chemistry applied more broadly and bolstered by established global norms and references could enable biologic discovery, early detection, risk stratification, and individualized therapeutic monitoring across a wide range of diseases.

## A recent discovery of an ancient phenomenon

In the late nineteenth century, chemists observed puzzling discrepancies in atomic weights among elements with similar chemical properties, and within the same groups of the periodic table. These anomalies suggested that atoms of a single element might not all be identical, though the concept remained undefined for some time. Building on this groundwork, Ernest Rutherford’s studies of radioactivity and atomic disintegration in the early 1900s provided critical insight into atomic transformations. In only two years, Rutherford and Frederick Soddy published a series of papers showing that radioactive atoms transform into new forms of matter and eventually into stable atoms [11, 12]. For this work, Rutherford (but not Soddy) was awarded the Nobel prize in 1908 for “his investigations into the disintegration of the elements, and the chemistry of radioactive substances”.

Soddy, however, continued his work into radioactivity and atomic transformation. He concluded that elements could share identical chemical properties yet differ in atomic mass. Therefore, he proposed that chemical identity depends on nuclear charge rather than weight. He also predicted that as a radioactive element, like uranium, decays to lead, the lead will have a different mass than ordinary lead. In a short Letter to Nature in 1913, he introduced the term *isotope* to describe variants that occupy the same position in the periodic Table [13]. The term isotope, meaning “same place”, was suggested to Soddy by Dr. Margaret Todd. Following Soddy’s discovery of isotopes came an intense decade of effort across laboratories that allowed scientists to see, understand,, and later use the phenomenon in far greater detail.

Influential first experimental evidence for isotopes came from the Brit Joseph John (JJ) Thomson. One of his experiments, using cathode rays in 1897, led to the discovery of the electron. Thomson had then turned to “positive electricity,” developing methods to separate positively charged ions by their mass-to-charge ratio. He built a so-called parabola where ions are separated by electric and magnetic fields, with their paths recorded on photographic plates. On a photographic plate in 1913, Thomson recorded the paths for neon gas with atomic weight 20 but also observed a faint trace corresponding to a mass of 22 – a neon isotope [14]. Soon followed a series of confirmative experiments that would reinforce the isotope concept, including at the Radium Institute in Vienna.

In Vienna, Stefanie Horovitz took on the task to test Soddy’s prediction that the element lead originating from uranium would be slightly lighter than ordinary lead. Using painstaking gravimetric analyses (i.e., dissolving rocks of ore, extracting, washing, filtering, dissolving and re-crystallizing repeatedly), Horowitz showed in 1914 that uranium-derived lead was indeed lighter, and therefore an isotope of lead [15]. When her lab director Otto Höningschmid left for a post in Munich, Horovitz stepped away from science and was later, sadly, deported to Treblinka [16]. At the time of her hard work to demonstrate the existence of isotopes, however, she was respected by Soddy and others in the community.

## From discovery to isotopic narratives

Experimental confirmation of isotopes came in 1919, when a previous assistant at the Cavendish Laboratory introduced the mass spectrograph. Thomson’s assistant Francis William Aston had joined the lab in 1910 to help build and refine the parabola apparatus. That experience let him push the technique further. His next instrument – the mass spectrograph – was capable of accurately measuring atomic masses with much higher precision and revealed that many elements are mixtures of isotopes [17]. The work that earned Aston the 1922 Nobel Prize paved the way for isotope analysis with steadily increasing precision [18]. Today, mass spectrometers can reach almost infinite resolving power for quantifying isotopic abundances, always with a trade-off of analytical time [19]. Even before the technique became widely accessible, two biochemists at Columbia University still found a way to use isotopes to probe how the body works.

Human physiology was the first field to apply stable isotopes when, in the 1930s, Rudolf Shoenheimer explored the idea that fat and proteins in the body are continuously produced and degraded. Together with David Rittenberg, they used the newly described heavy stable isotopes of water and hydrogen to trace metabolism [20, 21]. Before mass spectrometers were available, they detected heavy water through density measurements of purified tissue water. Thus, they fed rats and mice with food and water labelled with stable isotopes and measured in what tissues or waste these ended up. This work not only revealed the body’s “dynamic state” but also established isotope tracer biochemistry in medicine. While tracer biochemistry relies on mass balance and pathway connectivity, stable isotopes had one more level of utility once it became clear that physical and biological processes also fractionate them in predictable ways.

**Fig. 1:**
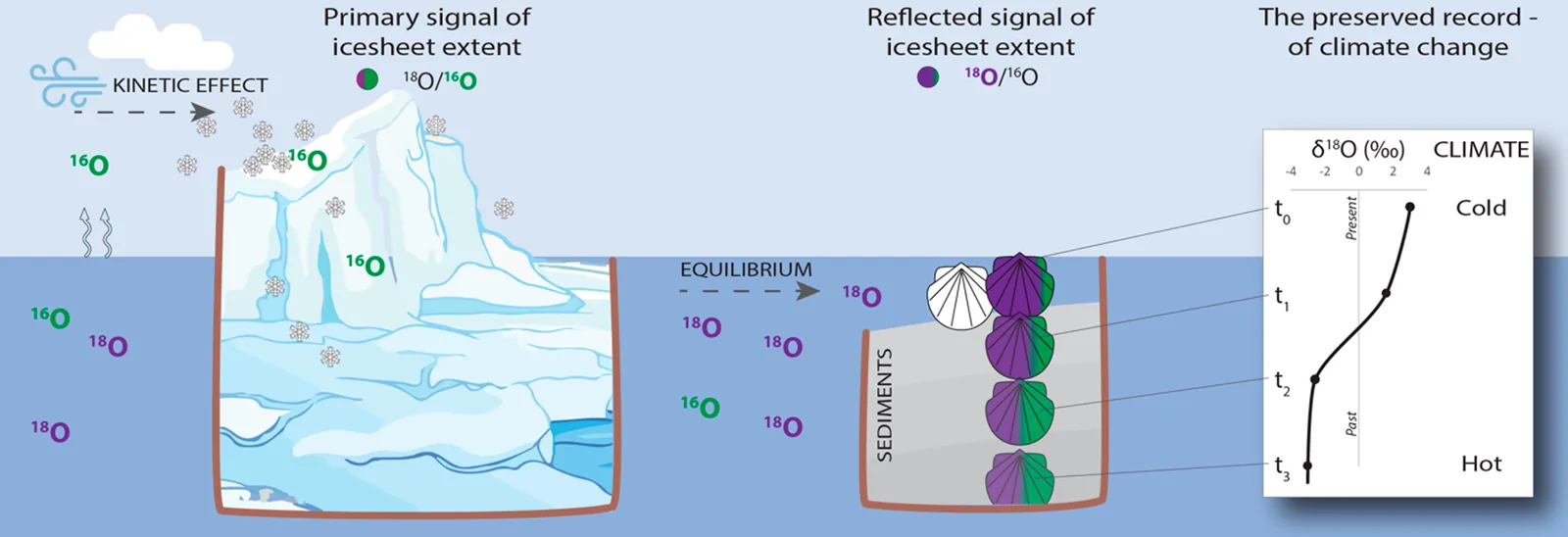
Schematic of how the lighter oxygen isotope (^16^O) is transported farther and is therefore preferentially stored in the terrestrial reservoirs, including the icesheet (a kinetic effect). Since the terrestrial icesheets grow during colder global climate, the remaining proportion of all oxygen isotopes is recorded in shells (of CaCO_3_) as these form in equilibrium with the ocean

The basis of isotope fractionation was established in the late 1940s when Harold Urey described why different masses of isotopes at process equilibrium result in uneven partitioning in chemical and biological systems [22]. Urey showed that small isotope mass differences alter bond vibrational energies and create predictable partitioning during reactions and phase changes (e.g., from liquid to solid states). He argued that at equilibrium, the heavier isotope tends to concentrate in the phase or molecular site where it is most strongly bound, i.e., where it is energetically most stable. For example, as oxygen changes state from liquid water (H_2_O) to the solid calcium carbonate mineral (CaCO_3_), the heavier oxygen isotope (^18^O as compared to ^16^O) will concentrate, i.e., fractionate, into the energetically more stable carbonate component. Since Urey also demonstrated, in theory, that this equilibrium fractionation of oxygen is a temperature dependent process, it led him to suggest that oxygen isotopes can reconstruct past temperatures [23]. In his example, assuming an animal makes shells of calcium carbonate in isotopic equilibrium with the water it lives in, measuring the shell’s oxygen isotope ratio allows you to infer the temperature of the water the animal inhabited. This represents the first use of isotopes as a paleothermometer (Fig. 1). In 1950, the temperature dependent direction of enrichment was corroborated experimentally by McCrae, also in Urey’s group, which ushered in the use of a paleotemperature scale in carbonate analyses [24] .

In parallel to equilibrium fractionation, the physical chemist Jacob Bigeleisen made it clear that isotopes also fractionate because of kinetic effects. Kinetic isotope effects state that lighter isotopes may, for example, react faster, diffuse easier, or be removed (distilled) in a process in a uni-directional way. In 1949, he formulated, together with Meyer, the quantitative theory of kinetic isotope effects (KIE) [25] and experimentally demonstrated it via isotope-dependent reaction rates to break carbon bonds [26]. After this, it took over the following decade until the geosciences started to extract this theory to describe, for example, natural variation in the ^18^O/^16^O ratio of waters and how isotope ratios in precipitation relate to climate [27, 28]. While kinetic effects are essential to explain environments that are, for example, diffusion-limited like sediments, the geosciences interpret isotope ratios using equilibrium fractionation as a baseline [29] .

To express such isotope ratios, results are reported using delta (δ) notation. Following Urey, small differences in isotope ratios are reported as delta values (δ) that express the isotope-number ratio of heavy to light isotope (R) in the sample normalized to the same R in a reference material (Eq. 1): δ = R_sample_ / R_standard_ – 1.

If Horowitz and Aston’s work confirmed that isotopes exist, medical researchers were the first to use labelled isotopes for tracing purposes. Once their predictable fractionation in biological and chemical processes became clear, however, stable isotope systematics was taken on by geoscientists in the 1960s and 1970s to reconstruct past state, infer origins, and more. This was also the time when mass spectrometers became accessible to a wider community. The material and extraction methods still varied, however, which led to joint efforts to align on reference material and scale. That collaborative era to standardize the method is a main reason why stable isotope chemistry is so comparable and, therefore, powerful across laboratories today.

## A community-built language made isotope data robustly comparable

Despite the technical advancements with more readily accessible mass spectrometers, it was clear that absolute isotope ratios were hard to measure with sufficient accuracy. McKinney et al.’s formalization of delta (δ) notation in 1950 helped launch the collaborative effort of a shared scale system that made isotope data comparable across laboratories, methods, and time [23, 30].

Yet, comparisons between laboratories were still hampered by the many extraction methods involved to produce the clean gases (e.g., CO₂ from carbonates) for analyses. Towards the end of the 1950s, the field had therefore started to share materials that served as practical ‘zeros’. This led to common reference scales anchored in shared standards such as PDB (Pee Dee Belemnite; δ^13^C), SMOW (Standard Mean Ocean Water; δ^18^O), CDT (Canyon Diablo Troilite; δ^32^S), and atmospheric nitrogen (δ^15^N) [31, 32]. To calibrate laboratory working standards to these shared one-point anchors efficiently allowed inter-lab and study comparisons, until reference materials started to run out.

In a subsequent ‘Vienna era’ from the 1960s, international bodies like IAEA (International Atomic Energy Agency, in Vienna), but also NIST (U.S. National Institute of Standards and Technology) and USGS (U.S. Geological Survey) began to establish replacement standards and enable their distribution across laboratories. Their reference materials created the scales used today, such as the Vienna-PDB (VPDB) for carbon, Vienna-SMOW (VSMOW) for water isotopes, and Vienna-CDT (VCDT) for sulfur. As instruments improved, laboratories also had to correct for scale compression and expansion, which drove the adoption of two-point or multipoint normalization. Because one-point correction alone could introduce differences in slope between laboratories, at least two certified reference materials with well-separated δ values are used to anchor both offset and scale.

Today, δ values are understood as scale-realized results that should be reported with reference to the international scale, the normalization approach, and uncertainty [33]. These developments over a near-century can be described as the evolution of a *method ecosystem*. At the core of this ecosystem is the definition of ‘fractional abundance’ by reference to an agreed-upon scale, the use of normalization that cancels instrument bias, and continuous intercomparisons [33, 34]. Few analytical methods achieve this degree of comparability, with pH and temperature among the closest parallels. That stable isotopes are used in fields from geology, ecology and hydrology to forensics and paleontology is a testament to its power to capture and compare chemical and biological states regardless of the analytical laboratory or timepoint [2]. These fields have the study of material sources and transformations in common, but also that they benefit from inexpensive and high-throughput ratio measurements. One field with a similar need is cancer detection, where signs of transformation could benefit from high-throughput and inexpensive analysis. So far, however, the use of stable isotopes in clinical medicine is limited.

## A breadth of applications

The importance of stable isotopes goes beyond that they can label and trace molecules, since they can also turn measurements into narratives of source, transformation, and constraint. Across disciplines, the most successful applications share the same logic, i.e., that isotope ratios become informative when they are interpreted against known fractionation rules, reference frameworks (standards, scale and normalization), and system dynamics. The examples below are therefore not a comprehensive inventory of applications, but illustrations of a broader principle – that isotopes add process-level information where concentration measurements alone are insufficient.

### Reconstructing past states along Earth and life history – geology and evolution

In the Earth sciences, stable isotopes became powerful when signals preserved in minerals, sediments, and shells could be read as records of past process [29]. While the early paleotemperature scale introduced by Urey and McCrea was fundamental for early paleoclimate work, stable isotope geochemistry has since become central to reconstructing Earth’s atmosphere, climate, habitability, and continental change through time [23, 24, 28, 35].

Different isotope systems record different aspects of this history. For example, the early build-up of an ozone layer is described via rare stable sulfur isotopes [36]. Prior to ozone layer formation, UV-light drove the mass-independent splicing of SO_2_ to SO in the atmosphere through photolysis, which led to an isotope signature in sulfur compounds, transferred from the air to the sediment, detected and analyzed eons later in the sedimentary rock record [37]. As oxygen built up in the atmosphere and the ozone layer formed, the window for UV light to drive this photolysis closed and the strong mass-independent sulfur signature disappears from the sedimentary rock record. In contrast, signatures produced by the microbial process of sulfate reduction, a mass-dependent process (a kinetic effect) remained to be detected in the record. In addition, the subsequent evolution of our planet’s oxygenation state can also be indirectly explored via sulfur isotopes (local indications added together) [38] but also e.g., chromium isotopes (soil mineral oxidation) [39] or molybdenum isotopes (global deep sea oxygenation) [40, 41].

Carbon isotopes occupy a particular place in geology since they connect metabolism (biological carbon fixation) to the sedimentary record and thereby allow for explorations of environmental and biological evolution [29, 42]. In living systems, carbon isotopes similarly distinguish dietary inputs; for example, δ13C can separate consumption of C3 versus C4 plants because these two photosynthetic pathways fractionate carbon differently. Carbon isotopes have also been used to infer very early evidence for biological carbon fixation, possibly from planktonic microorganisms in the water column (consistent with photosynthesis) [43]. Isotopes preserved in the layered rock record (stratigraphy) is a major tool for correlating basins across time. When integrated with paleontological data, such δ¹³C chemostratigraphy is key to resolve the timing and background of local and global evolutionary events, such as extinctions and diversification [44].

While geology show that isotope ratios can reflect and preserve process in minerals and sediments across deep time, biology demonstrate that the same logic applies to current and more recently historic living systems – where tissues record origin, diet, movement, and ecological interactions.

### Inferring origin and movement across space – biology, archeology, ecology, and forensics

In biology, isotope ratios are especially useful because living tissues integrate both source signals and physiological processing. As a result, isotope ratios can reveal provenance, ecological interaction, and life history [45].

Origin or provenance can be constrained by the spatial distribution of isotopes across a geographic area (isoscapes) that, later, is mapped in tissues. For example, feather δ²H can link birds to the environment in which the feathers formed. This can be combined with δ^18^O or δ¹³C that may reflect specific drinking-water, diet or habitat constraints. Classical work on migratory birds used feather δ²H to link wintering individuals back to broad breeding regions [46, 47]. The geographic migration, feeding patterns, or living conditions of prehistoric humans can be traced through strontium (^87^Sr/^86^Sr; reflecting geological substrate rather than mass-dependent fractionation) and oxygen (δ^18^O) isotopes in hard and soft tissues [48–50]. Building on this, nitrogen isotopes (δ^15^N) in bone collagen of prehistoric humans can resolve dietary composition and trophic level (e.g., vegetable or fish), especially when combined with δ¹³C to separate terrestrial vs. marine sources [51].

Microbial ecology via recordings of how elements such as carbon, nitrogen, and sulfur move through global biogeochemical cycles across the atmo-, bio-, litho- and hydrospheres. Nitrogen is a key example: because microbes control entry into and loss from the bioavailable nitrogen pool, nitrogen isotope systematics can record the balance of processes that shape productivity, food webs, and carbon uptake [52]. Although N is essential to life and common in the atmosphere, N_2_ is basically inert until microbes determine whether nitrogen gets into or out of the reactive pool (as nitrate or ammonium) and, therefore, becomes bioavailable [52]. The balance of reactive and inert nitrogen defines productivity in lake and ocean, carbon uptake, food webs and indirectly climate. And to decipher the relationship between all these processes and players, nitrogen isotope systematics are calibrated and, to the extent we understand this systematics, used as recorders of the ecology of the setting (local or global).

The biological applications extend into forensics, where the same tissue-level isotope signals are interpreted to make inferences about identity, origin, and movement that complement genetic and morphological approaches [53]. For example, in scalp hair, δ^2^H and δ^18^O reflect the isotopic composition of body water averaged over weeks to months. This signal is driven mainly by local drinking water and, to a lesser extent, by diet [54]. Since meteoric water isotope ratios vary in a structured way across regions, these patterns can be modelled as isoscapes and map geographic origin, migration or life-course exposure histories [55–57]. Approaches can also combine light bio-elements (δ^2^H, δ^18^O, δ13C, δ^15^N, δ^34^S) with heavier geo-elements such as radiogenic ^87^Sr/^86^Sr and, in some cases, lead [58, 59]. These approaches help identify unknown decedents, reconstruct mobility, and trace wildlife trafficking [60, 61].

The forensic community has contributed to the methodological framework established in the physical and Earth sciences, with a strong focus on calibration schemes, reference materials, and consistency across laboratories [62]. As an example, the human hair reference materials USGS42 and USGS43 were developed to anchor δ^2^H and δ^18^O measurements in keratin to the Vienna Standard Mean Ocean Water (VSMOW) scale [63]. When combined with shared protocols for sample cleaning, preparation, and data normalization, these materials support efforts to make hair-based provenance assessments reproducible and suitable for legal contexts [64]. Even so, interpretation remains probabilistic and constrained by data availability. For example, isoscape coverage is uneven worldwide and behaviors, such as bottled water consumption, can weaken links between hair and local tap water [58]. Therefore, strategies developed within the forensic sciences to address quality control offer a roadmap for future clinical applications of stable isotopes, where regulatory requirements are also strict.

### Resolving intake, exposure, and environmental interactions – epidemiology and public health

Once isotope ratios could be linked robustly to provenance, the same framework was extended to nutrition and exposure [65]. In health research, natural variation in carbon, nitrogen, and sulfur isotopes can distinguish among food sources and dietary patterns more objectively than self-report [56, 57, 66]. In public health, isotopes can also identify exposure sources, for example distinguishing lead derived from paint versus pipes [67, 68]. Keratinized tissues such as hair and nails are especially useful because they integrate intake and exposure over longer intervals than blood [69, 70]. The same principle underlies applications in doping control, falsified medicines, and food authentication [71–75].

In summary, isotope ratios are important tools for tracking many forms of nutrition, pollution, and environmental exposure [76–78]. These public-health uses bring the argument close to medicine: isotope ratios are valuable not only because they identify what entered the body, but because they retain information about how the body handled it. That shift — from exposure to physiology — is the clinical opportunity that can be explored further.

### Tracing function and detecting altered physiology – medicine

Medicine, then, is not an outlier to this story but its next logical step [79]. The field already adopted stable isotopes as safe tracers of function, but a larger promise may lie in revealing altered physiology directly through natural-abundance fractionation patterns [80].

Otto Warburg is famous in medicine for his work in the 1920s and 1930s to describe how cells consume oxygen and, by extension, how metabolism proceeds. Through continuous method development, he could detect minute gas changes above a living tissue slice and, eventually, also show that cancer cells rely on glycolysis even in the presence of oxygen [81]. However, it may at the time have been even more revolutionary to argue that tissues are not static, but instead dynamic by undergoing continuous synthesis and breakdown of compounds like lipids. This idea was advanced and tested by Shoenheimer, using labelled isotopes together with David Rittenberg who was recruited from Urey’s laboratory. Here, a trajectory began in which tracer approaches became important tools for the field of medicine.

Yet the clinical use of stable isotopes has largely remained within the tracer paradigm, even though the same fractionation principles that transformed the Earth sciences could, in principle, render naturally occurring isotope variation informative about disease mechanism. Today, ¹³C-labelled substrates are widely used to trace metabolic flux through glycolysis, the TCA cycle, and biosynthetic pathways, yielding insight into mitochondrial and cancer metabolism [4, 82, 83]. Tracers are also being explored to detect cancer cell metabolism, then detected in blood [84, 85]. Indirect tracing of disease via stable isotopes is also possible, such as tracing the microbial metabolism of the ulcer-inducing bacteria through a clinical breath test [80, 86]. In the ^13^C-urea breath test, urease-positive *Helicobacter pylori* hydrolyses labelled urea, where the resulting in ¹³CO₂ is detected in the exhaled breath [7]. Multiple other breath tests are being investigated for detection and monitoring of various cancers as well as metabolic disruptions such as diabetes and fat malabsorption [8, 87, 88].

Fractionation related to disease remains far less used than tracer approaches, yet it offers something tracers cannot: a read-out of altered physiology without any administered label. Although isotope effects – kinetic or equilibrium – are often small at the level of individual reactions, they accumulate across metabolic networks and become detectable in metabolites, tissues, and excreted products. Enzymatic bond-making and bond-breaking, pathway branching, lipid synthesis, transport, metal binding, and repeated cycles of synthesis and degradation can all reshape isotope distributions across molecular pools [9, 89]. Natural-abundance isotope measurements may therefore reveal altered physiology even in the absence of externally administered tracers, if source effects and clinical confounders are explicitly controlled.

**Fig. 2:**
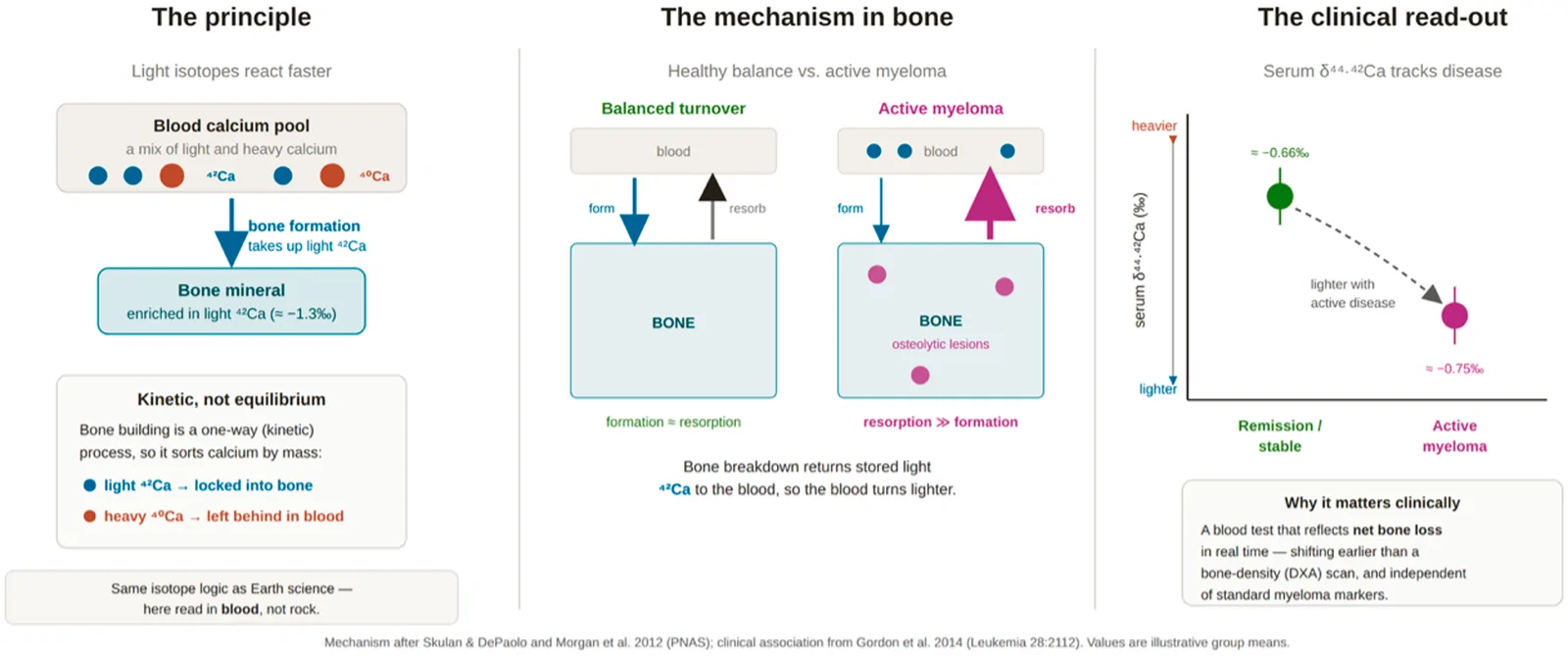
Calcium-isotope fractionation as a mechanistic biomarker of bone disease in multiple myeloma. (**A**) The principle of kinetic calcium-isotope fractionation during bone mineralization. (**B**) Mechanism of disrupted bone turnover in active disease. (**C**) Cclinical read-out in serum calcium-isotope composition

The available examples already span several isotope systems, biofluids, and tumor types. Serum calcium isotope fractionation (δ^44/42^Ca) tracks bone mineral balance in osteoporosis, chronic kidney disease, and multiple myeloma, where active disease shifts serum toward lighter values [90–92] (Fig. 2). Bone mineralization is a kinetic effectively one-way process that preferentially incorporates the lighter isotope, ^42^Ca, into the crystal lattice, leaving the residual blood pool enriched in heavier ^44^Ca, so that bone mineral is isotopically light relative to blood (Δ ≈ −1.3‰) by the same mass-sorting logic used in Earth-science depth profiles, here read in blood rather than rock [93, 94]. Under balanced turnover, bone formation and resorption are approximately equal and the serum calcium-isotope composition is stable; in active multiple myeloma, osteolytic resorption exceeds formation; the isotopically light calcium previously stored in bone is released back into the circulation, shifting the blood pool toward lighter values [94, 95]. Serum δ^44/42^Ca is accordingly lower in patients with active myeloma (mean ≈ − 0.75‰) than in those in remission or with stable disease (mean ≈ − 0.66‰), tracking disease activity earlier than bone-density (DXA) imaging and independently of standard biochemical markers [93–95]. In liver cancer (hepatocellular carcinoma), blood copper and sulfur isotope compositions differ from controls, which is consistent with copper redistribution into cysteine-rich proteins and tumor-associated sulfur pools [96, 97], and spatially resolved laser-ablation multicollector ICP-MS (LA-MC-ICP-MS) can now map copper isotope variation across tumor regions [98]. Breast tumors are enriched in light zinc isotopes relative to blood and healthy tissue, with metallothionein-linked zinc sequestration proposed as a mechanism [99, 100], while carbon and nitrogen isotope patterns in breast tissue and cell lines reflect altered lipid and nitrogen-retention metabolism [9, 101]. Bladder cancer studies combining plasma and red-blood-cell copper isotope composition with concentration and machine learning have achieved high classification performance [102], urinary zinc isotope and metallomic signatures have been reported in pancreatic and other secretory-organ cancers [103, 104], and C/N/S isotope differences distinguish endometrial tumor from healthy uterine tissue [105].

These differences are modest in absolute terms – typically a few tenths of a per mille (‰, i.e. parts per thousand as opposed to per cent, %). Serum δ^44/42^Ca differs by roughly 0.1‰ between active and inactive multiple myeloma [95]; serum δ^65^Cu is depleted by ~ 0.2–0.6‰ in several cancers, with tumor tissue ~ 0.5‰ heavier than adjacent normal tissue [106, 107]; and breast-tumor δ^66^Zn is ~ 0.15–0.9‰ lighter than surrounding tissue and blood [100]. On one hand, these signals can be seen as 1–2 orders of magnitude smaller than the natural-abundance gradients that Earth scientists sometimes encounter – for example the ~ 25‰ spread in plant δ¹³C between C_3_ and C_4_ photosynthetic pathways, or the tens-of-per-mille range in sedimentary δ^34^S [108]. On the other hand, their magnitude is closer to the transition-metal variation seen among terrestrial rocks (e.g. δ^56^Fe spanning ~ 2.5‰ in sediments) [109], and the variation measured within individual animals (where the within-organism δ^56^Fe, δ^65^Cu and δ^66^Zn span amplitudes similar to those in most terrestrial materials of roughly 1–3‰) [110]. Also, the magnitude is comparable to the fractionation within the human body itself (where δ^56^Fe differs by ~ 1–2‰ and δ^66^Zn by ~ 0.2‰ between serum and red blood cells) [111]. The clinical challenge is therefore twofold: to resolve and verify the magnitude of the disease-related signal and to, when possible, enhance the small signals since physiological and lifestyle variation of similar magnitude can perturb essential-metal isotope compositions [112]. With a signal that exceeds the ~ 0.05–0.1‰ precision of some mass spectrometry instruments, the analytical bar is attainable, especially if newly developed, pattern-based methods that trade single-measurement precision for greater dimensionality further strengthen the power to overcome such confounders.

Isotopic fractionation patterns may therefore provide a basis for detecting and monitoring malignancies. Yet, application in medicine remains limited, perhaps because of disciplinary barriers between medicine and chemistry, the short-term variability of liquid biopsies, or a mismatch between clinical priorities and available analytical technologies. Here, the forensic experience is instructive for the medical field. There, isotope data became actionable only once reference materials, standardized workflows, and probabilistic interpretive frameworks matured together.

Across these disciplines, the recurring value of stable isotopes is not merely that they can label matter, but that they preserve aspects of mechanism. In rocks, tissues, foods, and biofluids alike, isotope ratios become informative when they can be interpreted as the outcome of source mixing, fractionation, and system dynamics. Read this way, the diversity of applications is less a collection of case studies than evidence for a common analytical framework, one that medicine has only partly adopted. Why this framework is only partly adopted in medicine may relate to its specific needs and priorities.

Limitations, confounders, and clinical translation. Natural-abundance isotope ratios in human tissues and biofluids integrate source, physiology, and disease effects. Diet and geography can dominate light-element signals (for example, C_3_ versus C_4_ plant intake for δ¹³C; drinking-water patterns for δ²H and δ^18^O), and hospital diet changes can shift isotope composition during inpatient care [112]. Age, sex, menopausal status, renal and hepatic function, inflammation, medications, supplementation, microbiome composition, hydration, and sample-matrix differences (serum, plasma, red blood cells, urine, tissue) can all influence metal and light isotope distributions [112]. These factors are not arguments against isotope biomarkers, but they require matched disease controls, longitudinal sampling, paired concentration measurements, and explicit metadata capture before clinical claims are made. Operational barriers to clinical-scale metal-isotope analysis include instrument and consumable cost, specialist expertise, acid digestion, chromatographic isolation of individual analytes, handling of concentrated mineral acids, and limited throughput relative to routine clinical chemistry [113]. 

Positioning relative to established liquid-biopsy modalities. Medical oncologists will reasonably ask how natural-abundance isotope measurements compare with circulating tumor DNA (ctDNA), methylation assays, circulating tumor cells, extracellular vesicles, and conventional metabolomics or proteomics [114–118]. Those platforms address complementary questions: ctDNA reports tumor genotype; methylation assays support multi-cancer detection; circulating tumor cells capture intact-cell biology; extracellular vesicles carry nucleic acid and protein cargo; metabolomics surveys pathway state. Natural-abundance isotope ratios are unlikely to replace these modalities as stand-alone pan-cancer screens. Their more realistic niche is mechanism-linked adjunct information—metal redistribution versus concentration changes alone, bone mineral balance in myeloma, or paired isotope-concentration signatures integrated with machine learning; when incremental value over standard-of-care tests is demonstrated prospectively [102].

## Discipline-specific priorities and selection of technologies

Since Aston’s time of the early mass spectrometer, physicists and geoscientists have pushed isotope measurements toward ever greater analytical precision. Multi-collector mass spectrometers can now resolve extremely small differences in stable isotope composition, but that precision also comes with lengthy workflows involving digestion, chromatographic isolation of individual analytes, and long washout times [113]. Such protocols are suited to research questions in which sparse material, weak signals, and the need for cross-laboratory comparability justify analytical complexity.

Clinical medicine evolved under different constraints. In the clinic, methods are selected not only for analytical rigor, but also for speed, safety, throughput, and ease of interpretation. Stable isotopes therefore entered medicine mainly through tracer applications that met these practical demands. Breath tests for *Helicobacter pylori*, for example, provide rapid and interpretable functional readouts, using infrared light to quantify ^13^C in CO_2_, without requiring elaborate sample preparation or the highest-precision isotope platforms [7].

The same logic partly explains the appeal of stable-isotope imaging. Deuterium and ^13^C-based MRI avoid radioactivity, and hyperpolarized ^13^C-MRI can report metabolism in real time without the radiation burden of ^18^F-based PET [5, 119]. Deuterium metabolic imaging similarly offers a non-radioactive route to mapping glucose and glutamine metabolism in tumors [6]. These approaches succeeded not because they maximize isotopic precision, but because they answer clinically relevant questions in a practical format.

This difference in priorities may also explain why the Earth sciences developed a broader isotope “method ecosystem” earlier than medicine. Geological archives are often sparse, degraded, and biologically silent. For those reasons, isotope systematics may be the only route to reconstructing process. Medicine, by contrast, developed other high-performance molecular tools, from qPCR onward, that could interrogate biology rapidly and directly. As this revolution in biological tools took place, isotope methods in medicine remained largely within the tracer field.

That balance may now be shifting. Clinical applications may not require the full analytical precision demanded in isotope geochemistry if the goal is – not paleo-reconstructions with clues in partly degraded samples – classification, risk stratification, or treatment monitoring. High-throughput mass spectrometry could be isotope-capable workflows such as LC/GC-IRMS for compound-specific light-element isotope ratios; ICP-MS-based workflows for metal isotope systems including Ca, Cu, Zn, and Fe in biofluids, hair, or tissue digests; LA-ICP-MS/LA-MC-ICP-MS for spatially resolved elemental or isotope information; and LC/ESI-Orbitrap or FT-ICR-MS workflows that extract isotope-pattern or isotopologue features from metabolites, lipids or peptides. Combined with machine learning and multimodal clinical data, these workflows may extract clinically useful signal from isotope patterns even when individual measurements are less precise than those demanded in geoscience [120]. Instead, medicine may benefit from isotope analysis that is not maximally precise, but fit for purpose: robust, scalable, analytically validated, and informative enough for clinical decision-making. In essence, future medical applications that can analyze several dimensions of chemical data may need the mass spectrometry to be just ‘good enough’.

## Future opportunities in physiology and medicine

Stable isotope applications in medicine continue to expand, including personalized metabolic profiling and pharmacokinetic studies based on labelled compounds [121, 122]. For personalized metabolic profiling, isotope-labeled tracers could enable clinicians to tailor nutritional interventions, optimize drug dosing, and monitor disease progression without exposure to radioactivity. Together with advances in isotope-enriched pharmaceutical compounds, the tracking of drugs and pharmacokinetic could be enhanced.

For natural-abundance isotope biomarkers to become clinically useful, isotope measurements must be robust, scalable, and fast enough for clinical workflows. Importantly, they may not require the maximal precision sought in geochemistry if predictive performance can instead be achieved through multidimensional isotope features integrated with clinical and molecular data. Accurate precision can also be achieved by expanding on the dimensions of isotopic features that are studied. For that reason, the integration of stable isotope analysis with machine learning (ML) algorithms could contribute to fractionation-based applications in the medical oncology [123–126]. fractionate-based application in medical oncology [123–126]. The bladder cancer copper-signature study illustrates this direction by combining isotope composition, concentration, sample matrix, and random forest modelling. Future models should be trained in prespecified intended-use cohorts, locked before validation, and benchmarked against established clinical tests rather than optimized only for discovery performance. ML models can also learn non-linear relationships and interactions. When these algorithms can integrate stable isotope data with other clinical variables, imaging results, and genomic information, we will be able to construct patient profiles that support precision medicine strategies.

**Fig. 3:**
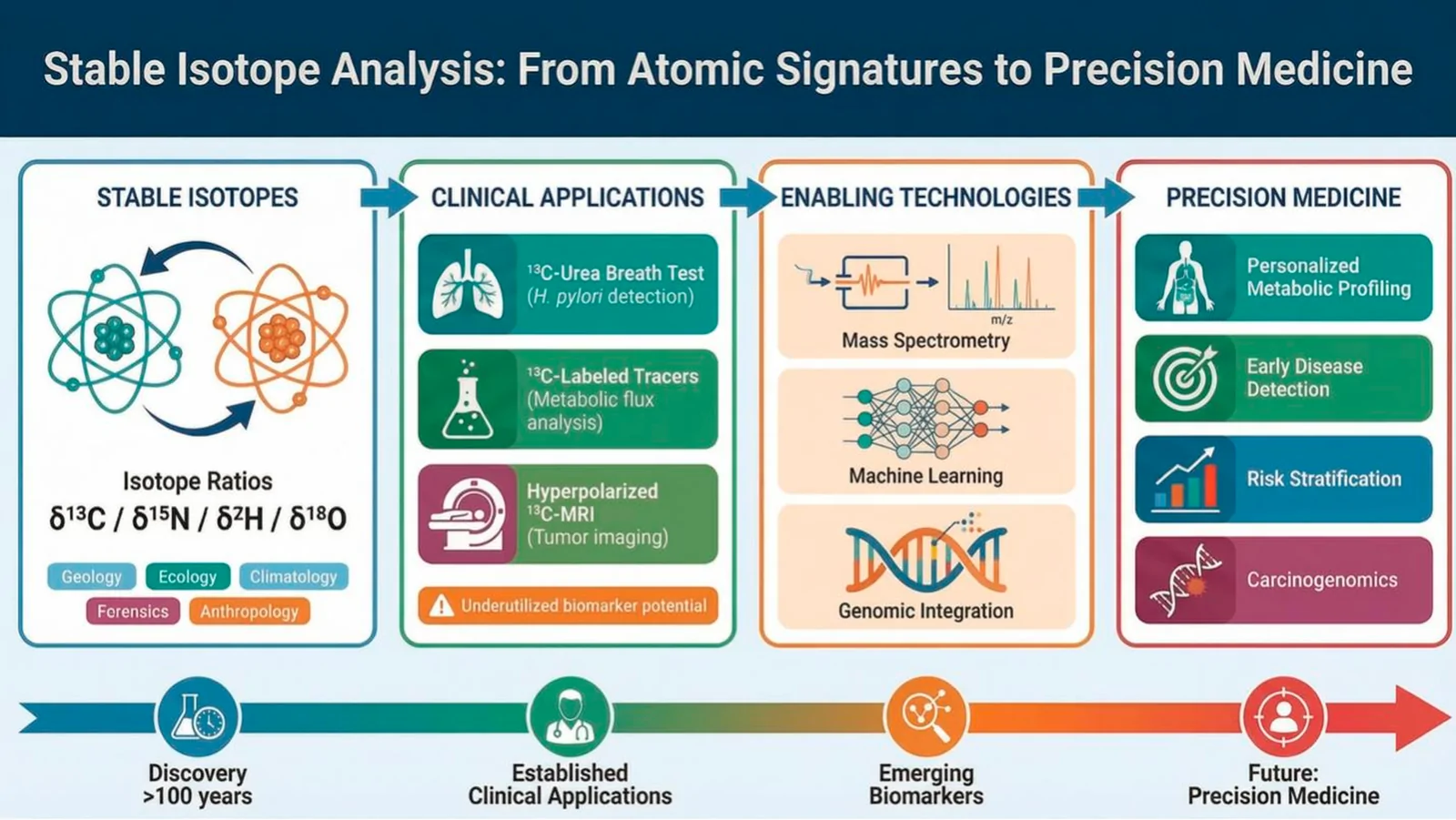
Stable isotopes in medicine. Stable isotope chemistry stands ready to move from the Earth sciences to the bedside, where it can enable biologic discovery, early detection, risk stratification, and individualized therapeutic monitoring across a wide range of diseases

Looking ahead, exposomics may be one of the intersections between stable isotope chemistry and data science. Stable isotope tools, together with complementary mass spectrometry-based exposure measurements, can help reconstruct dietary patterns, environmental inputs, xenobiotic metabolism, and endogenous pathway perturbations. Integrating whole genome sequencing (WGS) with exposomic profiling based on stable isotopes, other mass spectrometry exposure data, and machine learning could improve risk stratification and support more personalized prevention strategies (Fig. 3). In oncology, for example, integrating genomic susceptibility with exposure- and metabolism-linked isotope signals could provide a more mechanistic framework for actionable gene-environment hypotheses and enabling a new field of “carcinogenomics”. This oncology-focused review complements recent syntheses of natural isotope variation in disease and cancer omics [9, 112, 127], emphasizing translational requirements, confounder control, and comparison with established liquid-biopsy tools.

## Summary

Although recognized only a century ago, stable isotopes are as ancient as matter itself. If we learn to read them, they allow for processes and origin to be read from the same sample – something that concentrations measurements alone cannot do. While medicine pioneered to use stable isotopes to trace physiological function, their broader scientific history shows that they can reveal more. In the Earth sciences, isotope ratios became a robust measurement language for reconstructing source, transformation, and mechanisms across life’s ecology and evolution. These efforts have been possible thanks to collaborative efforts to share reference materials, scales and normalization method, so that isotope data could travel across laboratories and decades. While medicine adopted stable isotopes mainly for tracing and imaging, growing evidence shows that natural isotope variation in tissues and biofluids can also report altered physiology in disease.

We argue that advances in computation now make this broader isotope framework compatible with clinical settings, where robustness and throughput are key. In fact, further advances may be possible by combining current mass spectrometry and computational analysis with also genomic and clinical data. Then, some stable isotope measurements may become mechanism-linked biomarkers for early detection, risk stratification, therapeutic monitoring, and biological discovery.

## Data Availability

No datasets were generated or analysed during the current study.
